# First European *Erwinia amylovora* Lytic Bacteriophage Cocktails Effective in the Host: Characterization and Prospects for Fire Blight Biocontrol

**DOI:** 10.3390/biology13030176

**Published:** 2024-03-08

**Authors:** Elena G. Biosca, Ricardo Delgado Santander, Félix Morán, Àngela Figàs-Segura, Rosa Vázquez, José Francisco Català-Senent, Belén Álvarez

**Affiliations:** 1 Departamento de Microbiología y Ecología, Universitat de València (UV), 46100 Valencia, Spain; r.delgadosantander@wsu.edu (R.D.S.); felix.moran@ext.uv.es (F.M.); angela.figas@uv.es (À.F.-S.); rovazgar@alumni.uv.es (R.V.); mariabelen.alvarez@madrid.org (B.Á.); 2 Irrigated Agriculture Research and Extension Center, Department of Plant Pathology, Washington State University, Prosser, WA 99350, USA; 3 Departamento de Investigación Aplicada y Extensión Agraria, Instituto Madrileño de Investigación y Desarrollo Rural, Agrario y Alimentario (IMIDRA), 28805 Madrid, Spain

**Keywords:** phytopathogenic bacteria, biological control, phages, environmental samples, lysis, specificity, pear trees, loquats

## Abstract

**Simple Summary:**

*Erwinia amylovora* is a plant pathogenic bacterium responsible for fire blight, a devastating disease affecting economically important pome fruit trees such as apple, pear, and loquat. Chemical control of this pathogen has shown limited efficacy and poses risks to the environment and global health. The use of viruses that infect bacteria, named bacteriophages, can constitute an ecological alternative for fire blight control. This study aimed to search for *E. amylovora*-specific bacteriophages in Mediterranean environments where the disease was present. A collection of bacteriophages able to specifically infect and lyse *E. amylovora* was generated and characterized. The results allowed the description of the phages’ biology, interaction with the pathogenic bacterial host, and the selection of some bacteriophages for their potential application in controlling fire blight in the host. Assays in fruits revealed that the preventive application of some of the bacteriophages or their combinations delayed the onset of symptoms and reduced the severity of the disease. This study provides the first European *E. amylovora* phage cocktails effective in plant material. Our results are an example that environmental microorganisms can offer effective and sustainable natural solutions for the biocontrol of phytopathogenic bacteria to provide safe and healthy food.

**Abstract:**

Fire blight, caused by the plant-pathogenic bacterium *Erwinia amylovora*, is a highly contagious and difficult-to-control disease due to its efficient dissemination and survival and the scarcity of effective control methods. Copper and antibiotics are the most used treatments but pose environmental and human health risks. Bacteriophages (phages) constitute an ecological, safe, and sustainable fire blight control alternative. The goal of this study was to search for specific *E. amylovora* phages from plant material, soil, and water samples in Mediterranean environments. A collection of phages able to specifically infect and lyse *E. amylovora* strains was generated from former fire blight-affected orchards in Eastern Spain. Following *in vitro* characterization, assays in immature fruit revealed that preventively applying some of the phages or their combinations delayed the onset of fire blight symptoms and reduced the disease’s severity, suggesting their biocontrol potential in Spain and other countries. The morphological and molecular characterization of the selected *E. amylovora* phages classified them as members of the class *Caudoviricetes* (former *Myoviridae* family) and genus *Kolesnikvirus*. This study reveals Mediterranean settings as plausible sources of *E. amylovora*-specific bacteriophages and provides the first effective European phage cocktails in plant material for the development of sustainable fire blight management measures.

## 1. Introduction

*Erwinia amylovora* (Burrill) [1], a bacterial species belonging to the *Erwiniaceae* family [2], is the etiological agent of the highly infectious fire blight disease. This pathogen affects a large number of species of the *Rosaceae* family, including economically important fruit trees such as apple, pear, loquat, and quince, and also ornamental and wild plants [3,4]. The host’s shoots, leaves, flowers, fruits, and rootstock are susceptible to the disease, with infections significantly reducing fruit production and ultimately causing tree death. Under optimal conditions, fire blight outbreaks can destroy entire orchards in a single season, disrupting fruit production for years and leading to devastating economic losses in the areas where it is present [5,6,7].

The first report of fire blight dates back to 1780, discussing the detection of this disease in the Hudson Valley, in New York State (USA). Subsequently, it spread throughout North America and, in the 20th century, reached New Zealand, Egypt, and England through the trading of infected plant material, and, afterwards, the disease spread all over Europe and in the Mediterranean countries [4].

In Spain, *E. amylovora* was first detected in 1995, in the north of the country. Since then, some other outbreaks have been reported in northern, central, and eastern Spanish provinces. The current pest situation evaluated by the European and Plant Protection Organization (EPPO) is that the pathogen is present with restricted distribution and under eradication [8]. According to European Union (EU) regulations, in Spain and in the rest of the EU member states, *E. amylovora* is considered a Protected Zone Quarantine Pest (PZQP) [9]. The most effective approach to combat fire blight disease involves an integrated management encompassing prevention measures, early detection, and disease control strategies. Agrochemicals such as antibiotics (e.g., streptomycin) and copper compounds are usually the most effective form of control against *E. amylovora*. However, their persistent agricultural application has given rise to concerns regarding their long-term efficacy and sustainability. In the EU, the agricultural use of antibiotics is prohibited and that of copper-based compounds is highly restricted [10]. The selection of antibiotic-resistant and copper-tolerant strains [10,11,12,13,14], together with the negative effects of these compounds on the environment and human health, have led to a rising demand for safer and more sustainable agricultural practices [15,16,17,18,19].

The use of biological agents is a promising, environmentally friendly, and sustainable alternative to copper and antibiotics [20]. Currently, there are several commercial bioproducts based on bacteria (*Bacillus amyloliquefaciens*, *B. subtilis*, *Pantoea agglomerans,* and *Pseudomonas fluorescens*) or yeasts (*Aureobasidium pullulans*) with antagonistic activity against *E. amylovora.* However, their efficacy is variable and limited, and, in the case of *A. pullulans*, it is incompatible with the use of fungicides [21]. Bacterial viruses or bacteriophages (phages) may also be an option. Lytic phages are natural bacterial predators that can specifically infect and destroy their bacterial target, including pathogenic strains resistant to antibiotics and/or cupric compounds. Further, many of them are highly specific, usually infecting only a single pathogenic bacterial species without impacting the host plant’s beneficial natural microbiome or the surrounding environmental microbiota, thus being environmentally friendly [22]. 

However, the use of single bacteriophages can cause the appearance of resistant bacteria, but this can be solved by combining phages with different receptors of the target bacteria in cocktails which allow for reducing the emergence of phage-resistant bacteria as well as expanding the host range [23]. Using phages under field conditions faces other challenges, mainly the exposure to different types of environmental stresses, such as ultraviolet light, humidity, and temperature changes, as well as the survival of phages in the absence of their host bacteria [21,24]. Despite this, the bacteriophage-based treatment of bacterial plant diseases has received renewed interest in recent years, with several studies reporting promising control activities against important plant pathogenic bacterial species and several commercial phage-based products against some of them, including pathogens of the aerial part of the plant, such as *E. amylovora* [21,25,26,27,28,29,30,31,32,33,34].

*E. amylovora* phages can be isolated from infected plants and the surrounding soil and water [35,36,37,38], although their success in biocontrol is variable [39]. A key factor determining bacteriophage treatment efficacy, apart from their lytic nature, is their ability to persist on the phyllosphere. UV light and other environmental factors limit the success of phage application [21,24,40]. Some strategies to circumvent these challenges can be planning applications late in the day, working on protective formulations, and using carrier bacteria that minimize the effects of UV irradiation [23,41]. However, the isolation of phages adapted to the climate conditions of the locations where they will be applied, or at least similar to them, could help in maintaining high phage populations on plant surfaces and improve their efficacy.

As a preliminary step for the design of phage-based treatments against the fire blight disease, the goal of this study was to isolate and characterize *E. amylovora*-specific bacteriophages from Mediterranean regions in Spain, in which the disease was present. We tested the in vitro activity of different phage isolates against a collection of *E. amylovora* strains. Additionally, because *E. amylovora* is a quarantine organism in the EU and testing products in the field or even a regular greenhouse is not possible, we assessed the efficacy of preventive and control phage treatments using immature fruit. Based on their morphology, the bacteriophages isolated in this study were myoviruses. When applied preventively in immature fruits, i.e., before infection happens, the isolated phages demonstrated disease control capabilities. Our research addresses the critical need for effective and environmentally sustainable fire blight management strategies in Mediterranean and/or Mediterranean-like climate areas where existing disease control measures are limited. The evidence provided in our study constitutes the first steps toward designing a bacteriophage-based treatment to mitigate the impact of fire blight disease in these settings. Moreover, this work opens avenues for further investigation into the adaptability and efficacy of the isolated phages and phage cocktails in climatic conditions other than the Mediterranean. On top of that, the lytic phage cocktails assayed in this work constitute the first described in Europe with an effective biocontrol activity against *E. amylovora* in plant material, a matter which significantly adds valuable insights into the use of phage therapy in agriculture for plant disease control.

## 2. Materials and Methods

### 2.1. Bacterial Strains and General Growth Conditions

Spanish *E. amylovora* strains, UPN 527, IVIA 1554, IVIA 1526.6, IVIA 1614.1, IVIA 1614.2, and IVIA 1892.1, from different locations and host plant species (Table 1) were used as host strains for phage isolation and propagation. The strains were routinely cultured on the general media Luria Bertani Agar (LBA) [42] and Nutrient Agar amended with Sucrose and Yeast Extract (NASYE) [43] at 28 °C for 48 h. For bacteriophage isolation and amplification, liquid versions of the previous media (LB and NBSYE) were employed, also at 28 °C, with shaking at 150 r.p.m. 

The isolation of *E. amylovora* from plant material, and soil and water environmental samples (Table 1) as well as pathogen re-isolation from inoculated fruit were performed on a semi-selective CCT medium [44]. For the phage host range and specificity assays, a collection of 19 *E. amylovora* strains (Table 1) and 22 bacterial species were used (Table 2). In this case, *E. amylovora* strains were grown on NASYE and the other plant pathogenic bacteria on LBA, King’s B Agar (KBA) [45], or Yeast Extract Peptone Glucose Agar (YPGA) [46], depending on the bacterial species. The pure cultures of all the strains were cryopreserved at −80 °C in 25% (*v*/*v*) glycerol. The quarantine bacteria and samples were handled in a microbiology laboratory under Biosecurity Level 2 (BSL2) conditions.

### 2.2. Phage Enrichment, Isolation, Purification, and Amplification

Eight pear tree plots in the Valencian Community (Eastern Spain) with localized fire blight outbreaks were surveyed, and five of them were sampled in the late spring of 2018. The samples consisted of shoots, leaves, flowers, and fruits from symptomatic and asymptomatic trees as well as the soil below symptomatic trees and, when possible, from environmental water nearby the affected orchards. Once collected, the samples were placed into sterile bags and stored at 4 °C until use. 

For phage isolation, samples of 1, 5, and 10 g of each plant material type were washed with sterile distilled water for 1 h prior to being crushed with an antioxidant maceration buffer [47]. The volumes from the washing and crushing of the plant material samples were mixed in a ratio 1:1 with NBSYE 2X medium inoculated separately with different *E. amylovora* strains (UPN 527, IVIA 1554, IVIA 1526.6, IVIA 1614.1, IVIA 1614.2, IVIA 1892.1, and CFBP 1430) adjusted to an OD_600nm_ of 0.4 and incubated at 28 °C with shaking. Similarly, samples of 10 g or 10 mL of soil or water, respectively, were mixed with NBSYE 2X and inoculated as described above. The *E. amylovora*-specific phage content in the samples was enriched in a manner similar to Gill et al. [43] for 24 and 48 h at 28 °C and 150 r.p.m. Cell debris from cultures showing lysis was pelleted by centrifugation (10,000 r.p.m., 10 min) and filtered through filters with a 0.22 μm pore diameter to obtain clean phage suspensions. *E. amylovora*-inoculated media without samples were used as the negative lysis controls. 

For phage purification, filtered lysates were analyzed by serial decimal dilutions in an SM buffer (50 mM TrisHCl pH 7.5; 100 mM NaCl; 10 mM MgSO_4_ and 0.01% gelatin) and plated using the double-layer agar method. For this, 0.2 mL aliquots of bacterial culture (OD_600nm_ = 0.4) was mixed with 0.1 mL of phage filtrate dilution and 5 mL top agar (NASYE, 0.6% agar), poured onto NASYE plates and incubated at 28 °C for 24 h. 

After incubation, morphologically different plaque-forming units (PFU) were picked and purified with the respective host strain, and this process was repeated until obtaining identical plaques. 

The purified phages were propagated in NBSYE medium and titered as PFU/mL by means of the double-agar overlay assay. The pure phage suspensions were maintained for short periods at 4 °C and cryopreserved at −20 °C in 25% glycerol for longer periods.

### 2.3. Phage Host Range and Specificity

The host range of the isolated phages was performed by the spot test according to Born et al. [35], using a collection of Spanish and reference *E. amylovora* strains isolated from fire blight outbreaks in different host plants and locations, listed in Table 1. A specificity analysis of a selection of phages was also carried out by means of a spot test against bacterial strains representing species other than *E. amylovora,* including other phytopathogenic bacteria listed in Table 2. To accomplish so, melted top agar was mixed with 0.2 mL cultures (OD_600nm_ = 0.4) of the tested bacterial species and poured onto solid plates. Next, 5 μL of filtered pure phage lysates at about 10^8^ PFU/mL was spotted onto the top agar. Spots with cultures of the *E. amylovora* strains used for propagation were included as positive controls. Once the phage droplets were absorbed by the medium, the plates were incubated overnight at 28 °C. Areas of lysis below the phage spots were scored as positive, while those in which no changes were observed with the surrounding lawn were scored as negative. Additionally, the lytic activity of the selected phages was confirmed in liquid NBSYE medium. All the assays were repeated in independent experiments.

### 2.4. Bacteriophage-Based Biocontrol Assays In Vitro

#### 2.4.1. *E. amylovora* Biocontrol *In Vitro* by Single Phages

*E. amylovora* population dynamics were monitored in the presence of single phages. The growth dynamics of single phages were assayed by co-inoculation of the host strain and the selected phage in microtiter plates using a spectrophotometer plate reader (Tecan Infinite MNano, Männedorf, Switzerland) for 24 h at 28 °C and shaking, performing OD_600_ nm readings at regular intervals. Each well contained 0.2 mL of the host culture at around 10^6^ CFU/mL and phage at 10⁷ PFU/mL. Control wells containing only the NBSYE medium, the host strain, and phage suspensions alone were included. At least three replicates per phage and host strain were performed in at least two independent assays.

Comparisons between the growth of the *E. amylovora* strains alone and with individual phages were assessed during an incubation period of 23 h by calculating the areas under the curve (AUC) for each treatment. Then, the AUCs of the different treatments were compared using one-way Brown–Forsythe ANOVA tests (α = 0.05) designed for comparisons of groups with different SDs. Multiple comparisons between the controls and the treatments were carried out by Dunnett’s T3 post hoc analyses (α = 0.05).

#### 2.4.2. *E. amylovora* Biocontrol *In Vitro* by Phage Combinations

*E. amylovora* population dynamics were also monitored in the presence of phage cocktails or mixes. The growth dynamics of selected phage combinations or mixes (cocktails) were assayed as described above, by means of co-inoculation with single *E. amylovora* strains.

### 2.5. Bacteriophage-Based Biocontrol Assays Ex Vivo

#### 2.5.1. *E. amylovora* Biocontrol on Immature Fruits by Single Phages

Assays on immature fruit were carried out on loquats (*Eriobotrya japonica* cv. Tanaka) according to [20,48]. Briefly, 2.5–3 cm diameter fruits were surface-disinfected with 2% sodium hypochlorite, rinsed with sterile distilled water, and dried under the hood. Phage suspensions were prepared in an SM buffer at 10^8^ PFU/mL. *E. amylovora* suspensions were prepared in PBS at an OD_600_ of 0.2 (around 10^8^ CFU/mL) using NASYE plate cultures and diluted to 10^6^ CFU/mL. The disinfected fruits were wounded in three equidistant points with a sterile 100 μL pipette tip and used for fire blight preventive and control assays.

To test preventive treatments with bacteriophages, each wound was inoculated with 10 µL phage at 10^8^ PFU/mL (10^6^ PFU/wound) 24 h before inoculation with 10 µL of *E. amylovora* suspension at 10^6^ CFU/mL (10^4^ CFU/wound). For disease control (co-inoculation) experiments, 10 µL of phage plus 10 µL of *E. amylovora* were co-inoculated in each wound. Fruits inoculated only with PBS, SM, phages, or *E. amylovora* alone were included as the controls. The phages were assayed in three replicate fruits in at least two independent experiments. Challenged fruits were incubated at 28 °C in humid chambers, as described previously [20,48]. The fruits were monitored for the onset, incidence, and severity of fire blight symptoms. 

The efficacy of *E. amylovora* fire blight disease control by the tested phages as well as severity of symptoms were evaluated for 6 days after inoculation. Briefly, the disease control efficacy percentage (E) was determined as the E = (Ic − It/T) × 100, where Ic is the number of infected wounds in the phage-untreated *E. amylovora*-inoculated controls; It is the number of infected wounds in the fruit treated with both *E. amylovora* and phages; and T is the total wound numbers in each assay repetition. The disease severity after 6 days of incubation was determined after classifying the symptoms in five disease severity levels: 0 was the absence of symptoms; 1, sunken tissues, chlorinated tissue, or shade areas around the wound indicative of infection; 2, up to 2 mm wide necrotic lesions around the wound; 3, up to 4 mm wide necrotic lesions around the wound; 4, expanding necrosis (≥4 mm wide), usually reaching the peduncle and/or calyx areas of the fruit. 

The disease severity percentage (S) was calculated as S = $\left(\sum_{i=0}^{4}iNi/4T\right)\times 100$, where *i* is the severity index class, N*i* is frequency within the assay, T is the total number of wounds, and 4 represents the maximum severity index used for classification.

#### 2.5.2. *E. amylovora* Biocontrol on Immature Fruits by Phage Combinations

In this case, selected phage combinations or cocktails were used similarly, as explained above, for preventive and co-inoculation experiments. The phage cocktails assayed were mix 1 (UV_Eaϕ21, UV_Eaϕ24, and UV_Eaϕ28), mix 2 (UV_Eaϕ6, UV_Eaϕ21, UV_Eaϕ24, and UV_Eaϕ28), mix 3 (UV_Eaϕ6, UV_Eaϕ21, UV_Eaϕ24, UV_Eaϕ25, and UV_Eaϕ28), and mix 4 (UV_Eaϕ6, UV_Eaϕ21, UV_Eaϕ24, UV_Eaϕ25, UV_Eaϕ27, and UV_Eaϕ28). The fruits were inoculated, incubated, monitored, sampled, and processed as described above.

A statistical analysis of the data obtained from biocontrol assays was performed using GraphPad Prism version 9. 

### 2.6. Phage Morphology

Phage virions from 0.22 μm filtered freshly prepared phage lysates (about 10^10^ PFU/mL) obtained as mentioned above (Section 2.2) were prepared for transmission electron microscopy (TEM) as described by Biosca et al. [49]. In short, 5 μL of lysates was absorbed on fresh formvar and carbon-covered grids for 1 min. The excess of sample was removed, and the grids were stained with 1% of phosphotungstic acid (pH 7.0) for 1 min and air-dried. Electron microscopic visualizations of the phage virions were performed using the TEM Hitachi HT7800 operated at 120 kV and with a 20 Mpx CMOS EMSIS XAROSA digital camera, at the Central Service for Experimental Research (SCSIE) facility (Universitat de València, Burjassot, Spain). The phage virion dimensions (head diameter and tail length) were determined on micrographs with the ImageJ software version 1.53m [50] in at least 10 viral particles from each phage.

### 2.7. Phage Molecular Characterization

#### 2.7.1. Phage Genome Sequencing

DNA extraction from selected phages was performed according to Biosca et al. [49]. Shortly, bacterial nucleic acids in filtered phage lysates were eliminated by treatment with DNase and RNase for 1 h at 37 °C, followed by heat inactivation with EDTA for 10 min at 37 °C. 

Phage DNA was isolated with the NucleoSpin^®^ Plasmid isolation kit (Macherey-Nagel, Düren, Germany), following the protocol for low-copy plasmid isolation. 

Total DNA quantification was performed using a Qubit 4^TM^ Fluorometer (ND-2000, ThermoFisher, Wilmington, DE, USA) with dsDNA HS Assay KitTM. DNA libraries were made with Nextera XT Library Preparation Kit (Illumina, San Diego, California, USA) following the manufacturer’s instructions. Sequencing was carried out using the Illumina MiSeq platform with 2 × 250 bp paired-end sequencing (Illumina, USA). Raw data read outputs were subjected to quality control, trimming, and de novo assembly with the CLC Genomics Workbench 10.1.1 software (QIAGEN, Hilden, Germany). De novo contigs were analyzed by BLASTn and BLASTx [51].

#### 2.7.2. Phage Genome Annotation

Open Reading Frames (ORFs) search in *de novo* assembly contigs was performed with Geneious Prime^®^ 2023.2 based on nucleotide similarity with the RefSeq genome NC_041978. In addition, genome annotations were confirmed by RASTtk [52], and all the annotated ORFs were reviewed and refined through database searches using BLASTp [51].

For the specific search of proteins in the newly assembled genomes, phage proteins were downloaded from NCBI (20,006 Endolysins, 1331 Holins and 608 Depolymerases, accessible in December 2023) and were compared using tBLASTn against the newly assembled genomes, using the Geneious Prime^®^ 2023.2 software. All the annotated genomes were deposited in the NCBI database (PP079182, PP079183, PP079184, PP079185, PP079186, and PP079187). 

The prediction of lytic or lysogenic lifecycle was performed with the tool PhageAI [53], available online [54] with code https://github.com/phageaisa/phageai (accessed on 29 February 2024)

#### 2.7.3. Phage Phylogenetic Analysis

Phylogenetic analyses were performed with the whole genomes of the six Spanish phages and a selection of twenty-eight RefSeq viral genomes belonging to the subfamily *Ounavirinae,* native to China, Lithuania, Vietnam, Canada, South Korea, Spain, and the USA. 

Accurate complete-genome alignment was performed with MAFFT V.7 [55]. The phylogenetic tree was reconstructed in MEGA X [56], using the maximum likelihood algorithm, supported using 500 bootstrap replicates, and selecting the best substitution model computed (GTR + G) implemented. In addition, a proteomic tree was constructed from a selection of reference genomes with the ViPTree web server [57] based on normalized tBLASTx scores. For the viral proteomic tree figure, a Newick format file was downloaded from the VipTree web server and visualized in the Geneious Prime^®^ 2023.2 software.

## 3. Results and Discussion

### 3.1. Isolation of Mediterranean E. amylovora-Specific Bacteriophages Linked to Presence of Symptomatic Plant Material

A collection of 124 bacteriophages was isolated from four out of the five analyzed plots. Most isolated phages came from plant material (79.8%). Specifically, all the positive phage isolations came from symptomatic pear tree samples. The next main source of *E. amylovora* bacteriophages was the soil underneath the affected pear trees (16.9% phages), followed by environmental water near the affected trees (3.2% phages). Symptomatic plant material and the soil beneath diseased trees are frequently reported to be good sources of *E. amylovora* bacteriophages [36,43,58]. 

The isolation of bacteriophages from water sources in fire blight-affected areas might indicate the presence of *E. amylovora* cells in the water. Under laboratory conditions, the pathogen can survive in distilled water, mineral water, rainwater, and river water for variable periods depending on the incubation temperature, the presence of microbiota, the type of water, etc. [59,60,61]. However, our attempts to isolate the pathogen in the water samples where the phages had been found were unsuccessful. Although the transitory presence of *E. amylovora* cannot be discarded, it is worth mentioning that phage concentrations are usually ten times higher than those of their bacterial target in the environment [62,63]. Another possibility, apart from a native higher survival capability, could be that the isolated phages are also natural predators of other host species present in the water. Indeed, wastewater has been reported as a source of bacteriophages against *E. amylovora* and other plant pathogens [64,65].

### 3.2. Mediterranean Bacteriophages Active against E. amylovora Strains from Different Locations and Hosts

All the isolated bacteriophages showed lytic activity against different *E. amylovora* strains, including Spanish, French, Serbian, and American strains isolated from pear, apple, and ornamental and wild host species of the genera *Cotoneaster*, *Crataegus*, and *Pyracantha*. Among the collection of bacteriophages isolated initially, 28 showed lytic activity against 58–100% of the tested *E. amylovora* strains, according to a spot assay (Table 3). Information about the origin of the 28 selected bacteriophages, that is, from UV_Eaϕ1 to UV_Eaϕ28 (hereafter abbreviated as ϕ1 to ϕ28), can be found in Supplementary Table S1. 

Despite the high genome homogeneity of the *E. amylovora* species, bacteriophages active against a set of *E. amylovora* strains usually lack activity against a more or less considerable percentage of the tested strains [24,36,66,67]. 

These differences have been associated with factors such as the geographical origin of the host strains and their relative amylovoran production. The capacity of bacteriophages to infect different *E. amylovora* strains also probably correlates with differences in the host strain CRISPR system, the receptors for phage infection [67], and other unexplored resistance mechanisms in this pathogen.

Specificity tests with the selected 28 bacteriophages showed no lytic activity against bacterial species other than *E. amylovora*, including plant pathogens and other Gram-positive and Gram-negative species from different countries and sources (Table 3).

**Figure 1:**
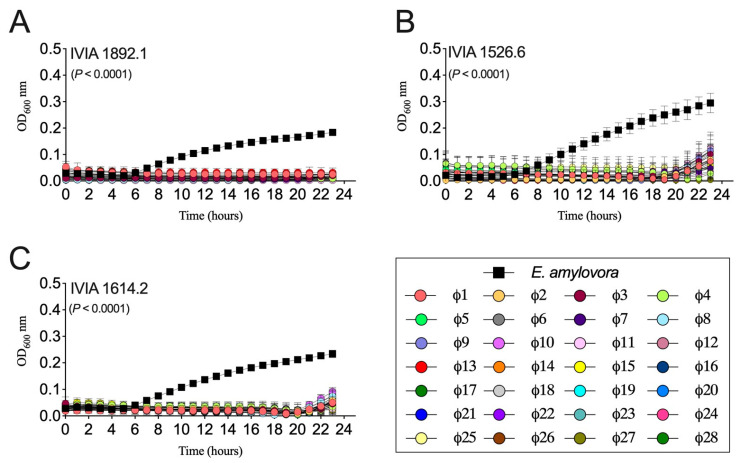
*Erwinia amylovora*–phage interactions in liquid NBSYE medium. Graphs show the growth of Spanish *E. amylovora* strains (**A**) IVIA 1892.1, (**B**) IVIA 1526.6, and (**C**) IVIA 1614.2 alone (squares) and in a co-culture with Mediterranean *E. amylovora* phages ϕ1 to ϕ28 (circles), assayed individually. Data points are the average values of triplicate experiments, and the error bars show the standard deviation (SD). The *p* values in each chart show the differences between the areas under the curve (AUC) of the controls (*E. amylovora* grown alone) and treatments (*E. amylovora* plus individual phages) based on a Brown–Forsythe ANOVA test.

Assays in liquid culture confirmed the lytic activity of the 28 bacteriophages against a selection of nine Spanish *E. amylovora* strains. Some phages that apparently did not lyse certain *E. amylovora* strains in spot assays showed high activity against the same strains in a liquid medium. As an example, phages ϕ15, ϕ20, ϕ22–24, and ϕ27–28, which did not show lytic activity against *E. amylovora* IVIA 1892.1 in a spot assay (Table 3), were able to completely lyse populations of the same strain for 20–23 h in a liquid medium (Figure 1A). Experimental method-dependent differences in the lytic activity of bacteriophages have been reported by different authors [67,68]. The reasons behind these differences can be explained by the diversity of phage resistance mechanisms and the infection steps each method favors, can detect, and/or relies upon [69]. Factors affecting host cell growth, production of exopolysaccharides, etc., may also contribute to variability in the results depending on the methodology and conditions employed.

All the selected Mediterranean phages exerted control of *E. amylovora* populations in liquid cultures, efficiently reducing the bacterial populations to the initial numbers for 15–23 h, depending on the tested host strain and phage (Figure 1). The most sensitive strain was IVIA 1892.1, which was lysed by all the bacteriophages with barely any changes in the OD_600nm_ values from time 0 to 20–23 h (Figure 1A). In the remaining *E. amylovora* strains, towards the last 3–4 h of the experiment, a slight increase in bacterial populations was observed while being treated with different phages (Figure 1B,C), similar to other works [70,71]. This can be explained by the selection of phage-resistant host cell subpopulations under the assayed in vitro conditions [22,63,72,73]. 

Regardless of the assayed host strain and phage, the OD_600nm_ values within the assayed period (23 h) were significantly lower (*p* < 0.0001) in the *E. amylovora* cells treated with individual bacteriophages than in those grown alone, as revealed by the AUC comparison analysis (Figure 1). 

### 3.3. Designed Phage Mixes Control E. amylovora Populations under In Vitro Conditions

In this study, four different phage mixes with combinations of bacteriophages ϕ6, ϕ21, ϕ24, ϕ25, ϕ27, and ϕ28 were designed. These phages were selected based on their origin (plant material, soil, and water) (Supplementary Table S1) and their lytic activity against *E. amylovora* strains from different geographical locations and hosts (Table 3). Again, those phages selected that did not appear to lyse some strains of *E. amylovora* in the spot test did show lytic activity against the same strains in the liquid medium (Table 3). The phage compositions of the four mixes were as follows: MIX 1, phages ϕ21, ϕ24, and ϕ28; MIX 2, phages ϕ6, ϕ21, ϕ24, and ϕ28; MIX 3, phages ϕ6, ϕ21, ϕ24, ϕ25, and ϕ28; and MIX 4, phages ϕ6, ϕ21, ϕ24, ϕ25, ϕ27, and ϕ28. 

**Figure 2:**
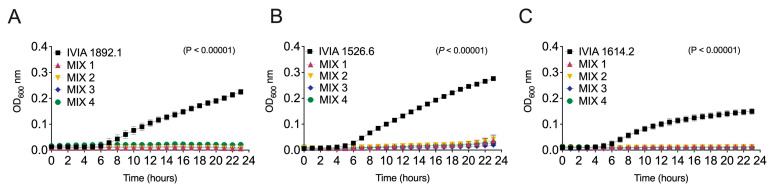
*Erwinia amylovora*–phage interactions in liquid NBSYE medium. The graphs show the growth of Spanish *E. amylovora* strains (**A**) IVIA 1892.1, (**B**) IVIA 1526.6, and (**C**) IVIA 1614.2 alone (black symbols) and with the Mediterranean *E. amylovora* phage mixes (colored symbols). The data points are the average values of sextuplicate assays, and the error bars show the standard deviation (SD). The *p* values in each chart show the differences between the areas under the curve (AUC) of the controls (*E. amylovora* grown alone) and treatments (*E. amylovora* plus phage mixes) based on a Brown–Forsythe ANOVA test.

Figure 2 shows the activity of the four phage mixes against three Spanish *E. amylovora* strains from different locations and hosts. In all cases, the phage mixes reduced the *E. amylovora* populations to around their initial levels throughout the assayed period (*p* < 0.0001). Moreover, the slight increase in *E. amylovora* populations seen at the end of the experimental period when using individual phages (Figure 1) was either suppressed, as in the case of strain IVIA 1614.2 (Figure 1C *vs*. Figure 2C), or reduced compared to when many of the assayed individual phages were used against strain IVIA 1526.6 (Figure 1B *vs*. Figure 2B). 

The use of phage cocktails instead of individual phages has some advantages. The co-evolution of phages and pathogens in the same environments may lead to the accumulation of different phage-resistance mechanisms in the host cells. These mechanisms may target phage adsorption onto the host cell’s surface and other stages of the phage infection cycle [73,74]. The emergence of phage-resistant bacteria can be minimized by using optimized mixtures of bacteriophages with different mechanisms of action and/or adaptation to different conditions. The range of virulence genes and degrees of environmental tolerance of the phages contained in a phage cocktail may also help compensate for each of the phages’ limitations, enhance their abilities, and/or improve their global efficacy under varied conditions [27,75,76]. The phages in a mix might also use different host cell surface receptors and strategies to overcome bacterial defense mechanisms against viral nucleic acids, like CRISPR-Cas systems, restriction-modification systems, etc. [77]. This makes it harder for bacteria to develop multiple resistance mechanisms simultaneously, contributing to the control efficacy of the mix. Finally, cocktails of phages with different host specificities may also help broaden the range of target bacterial strains [78]. 

### 3.4. Preventive Application of Phage Mixes Increases Disease Control Efficacy and Reduces Symptom Severity with Respect to Individual Phages in Detached Fruit Assays

**Figure 3:**
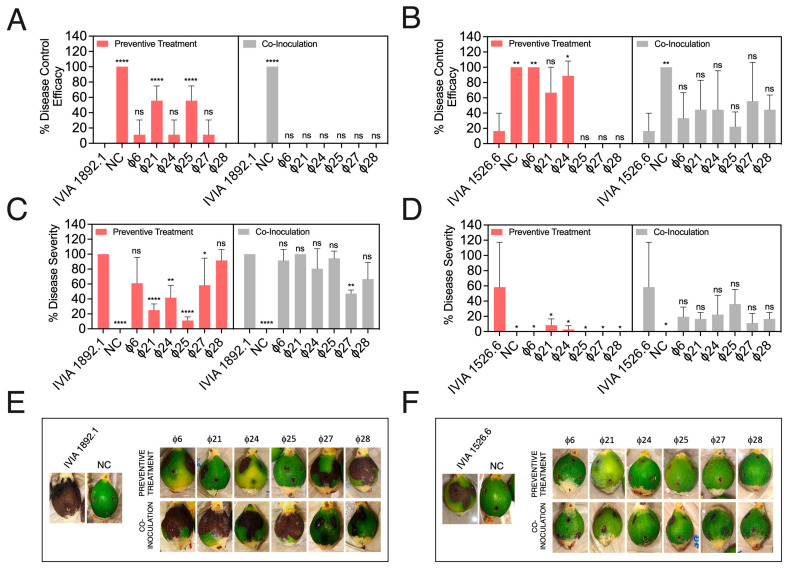
Biocontrol activity of a selection of six individual Mediterranean *Erwinia amylovora* phages against *E. amylovora* strains IVIA 1892.1 (**left**; **A**,**C**,**E**) and IVIA 1526.6 (**right**; **B**,**E**,**F**) in detached, green loquat fruit. In the preventive treatments, the selected phages were applied individually 24 h before *E. amylovora* inoculation. In the co-inoculation assays, both *E. amylovora* and individual phages were applied simultaneously. (**A**,**B**) Disease control efficacy (%), (**C**,**D**) disease severity (%), and (**E**,**F**) representative images of inoculated fruits after a 6-day incubation period at 28 °C. The columns represent the average values of triplicate assays, and the error bars depict the standard deviation (SD). The asterisks show significant differences among the fruits inoculated with *E. amylovora* alone, those treated with the pathogen plus each phage, and the negative controls (NC); ns stands for not significant; *, *p* < 0.05; **, *p* < 0.01; ****, *p* < 0.0001, assessed using Dunnett’s multiple comparison tests (α = 0.05); ns stands for not significant.

Figure 3 shows the individual phage activities against the Spanish *E. amylovora* strains IVIA 1892.1 (Figure 3A,C,E) and IVIA 1526.6 (Figure 3B,D,F). A two-way ANOVA analysis revealed that, regardless of the assayed *E. amylovora* strain, both the timing of phage application (preventive *vs*. co-inoculation) and the treatment type (with or without the different phages) had a very significant effect on the outcome (in both cases, *p* < 0.0001). 

In experiments with the ϕ6, ϕ21, ϕ24, ϕ25, ϕ27, and ϕ28 phages and the *E. amylovora* strain IVIA 1892.1, only phages ϕ21 and ϕ25 provided a significant control efficacy of around 56% but only when applied preventively (*p* < 0.0001) (Figure 3A). This application method not only improved the disease control efficacy percentage but also significantly reduced the disease severity in the inoculated fruits. Preventive treatments with phages ϕ21 and ϕ25 and also ϕ24 and ϕ27 revealed disease severity percentages of 11.1% (*p* < 0.0001), 25.0% (*p* < 0.0001), 41.7% (*p* = 0.0014), and 58.3% (*p* = 0.0358), compared to the 100% observed in the fruit inoculated with *E. amylovora* alone (Figure 3C,E). In most cases, the bacteriophage treatments were ineffective when co-inoculated with *E. amylovora* (Figure 3A,C,E). One exception was the treatment with ϕ27, which reduced the disease severity by around 53% (*p* < 0.0043) with respect to treatments with *E. amylovora* alone (Figure 3C,E). 

The Spanish *E. amylovora* strain IVIA 1526.6 was less virulent than the strain IVIA 1892.1 (Figure 3), but, overall, we observed similar trends with both strains. Preventive phage application provided an overall better result in controlling the incidence and the severity of symptoms than when co-inoculated with *E. amylovora* (*p* < 0.0001). In this case, the treatments with phages ϕ6, ϕ24, and ϕ21 conferred 100.0%, 89.0%, and 66.7% disease control efficacy, respectively, when applied 24 h before *E. amylovora* inoculation. However, only the results with phages ϕ6 and ϕ24 were statistically significant (*p* ≤ 0.0273) (Figure 3B). Regarding the disease severity, preventive phage application avoided symptom development (phages ϕ6, ϕ25, ϕ27, and ϕ28) and/or provided significant symptom reduction (phages ϕ21 and ϕ24), all these effects being statistically significant (*p* ≤ 0.0107) (Figure 3D,F). Co-inoculation treatments with *E. amylovora* IVIA 1526.6 had no significant effects on disease control efficacy (Figure 3B). Although all the co-inoculated fruits showed clear reductions in disease severity, none of the observed effects resulted in statistical significance (*p* ≥ 0.1231) (Figure 3D,F).

**Figure 4:**
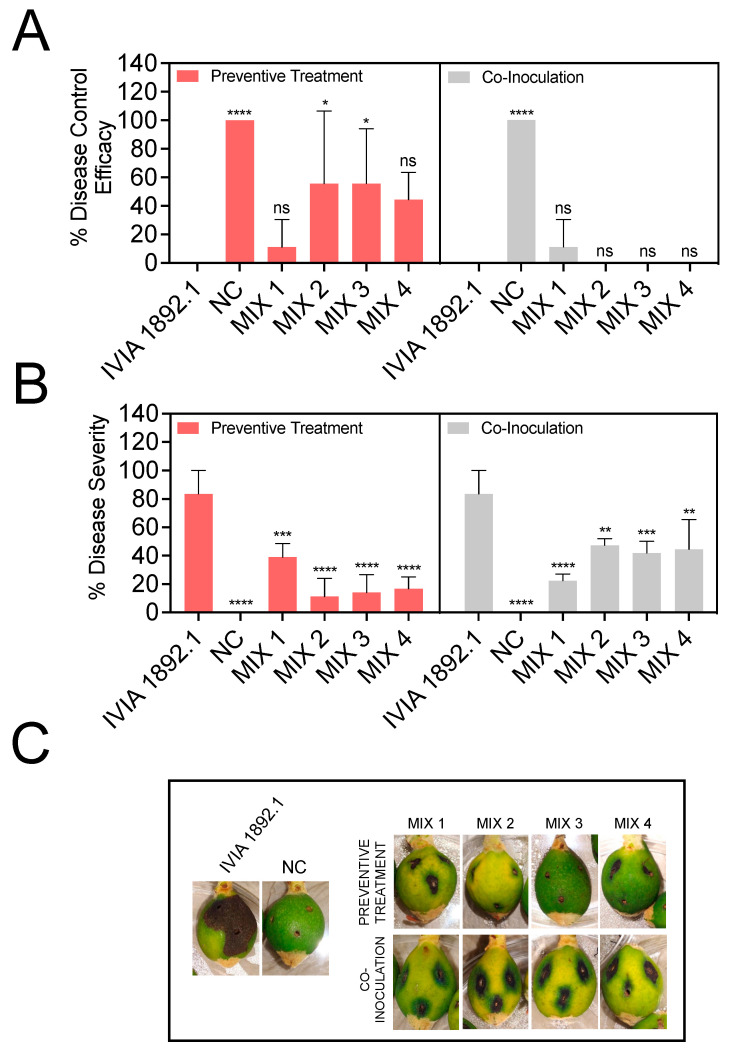
Biocontrol activity of a selection of four Mediterranean *Erwinia amylovora* phage cocktails (MIX 1–4) against *E. amylovora* IVIA 1892.1 in detached, green loquat fruit. In the preventive treatments, the phage mixes were applied 24 h before *E. amylovora* inoculation. In the co-inoculation assays, both *E. amylovora* and the phage mix were applied simultaneously. (**A**,**B**) Disease control efficacy (%) and disease severity (%), respectively. The columns represent the average values of triplicate assays, and the error bars depict the standard deviation (SD). The asterisks show significant differences among the fruits inoculated with *E. amylovora* alone, those treated with the pathogen plus the indicated phage mix, and the negative controls (NC), assessed using Dunnett’s multiple comparison tests (α = 0.05); ns stands for not significant; *, *p* < 0.05; **, *p* < 0.01; ***, *p* < 0.001; ****, *p* < 0.0001. (**C**) Representative images of inoculated fruits at the end of the assays (6 days at 28 °C).

The performance of the bacteriophages improved when mixed in cocktails (Figure 4). Like the application of individual phages, the co-inoculation of the phage mixes plus *E. amylovora* did not significantly improve the disease control efficacy (Figure 4A). However, the preventive use of the bacteriophage mixes 2, containing phages ϕ6, ϕ21, ϕ24, and ϕ28, and 3, containing the aforementioned phages plus ϕ25, provided a significant increase in the disease control efficacy of around 56% (*p* ≤ 0.0136). The most significant effects of the phage mixes were linked to the reduction in symptom severity (Figure 4B,C). In this case, both the preventive application and the co-inoculation of the phage mix with the pathogen significantly reduced the disease severity. However, the best results were obtained after the preventive application of the phage mix (Figure 4B,C). For instance, the bacteriophage mixes reduced symptom severity from 83.3% in the fruit inoculated with *E. amylovora* IVIA 1892.1 alone to 11.1–38.9% when applied 24 h before *E. amylovora* inoculation (*p* ≤ 0.0004) and 22.2–47.2% when co-inoculated with the pathogen (*p* ≤ 0.0040). 

**Figure 5:**
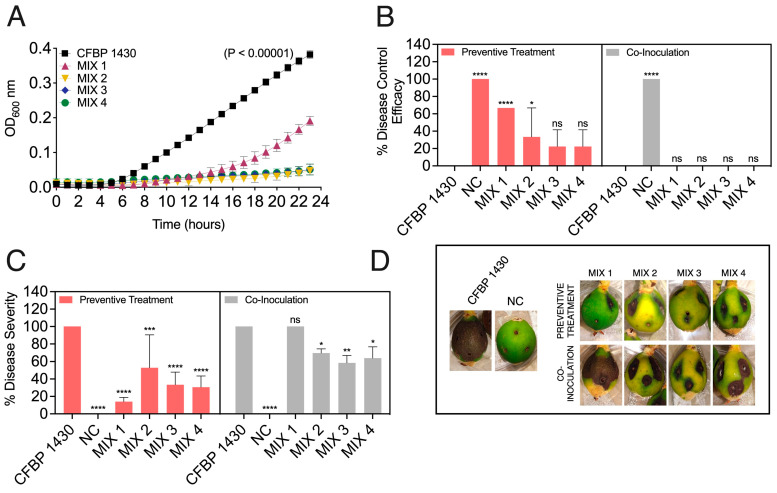
Performance of four Mediterranean *Erwinia amylovora* phage cocktails (MIX 1–4) against the French *E. amylovora* reference strain CFBP 1430. (**A**) Effect of the phage mixes in *E. amylovora* growth in a liquid medium. Each data point shows an average of six replicates, and the error bars denote the standard deviation (SD). The *p* value indicates the significance of differences between the AUCs obtained by *E. amylovora* grown alone (black symbols) and in a co-culture with the phage mixes (colored symbols), assessed by a one-way Brown–Forsythe ANOVA test. (**B**–**D**) Biocontrol activity of the phage mix in detached, green loquat fruits after 6 days at 28 °C. In the preventive treatments, the phage mixes were applied 24 h before *E. amylovora* inoculation. In the co-inoculation assays, both *E. amylovora* and the phage mix were applied simultaneously. (**B**,**C**) Disease control efficacy (%) and disease severity (%), respectively. The columns show the average results of an experiment performed in triplicate, and the error bars are the SD. The asterisks indicate statistically significant differences among the fruits inoculated with *E. amylovora* alone, those treated with the pathogen plus the indicated phage mix, and the negative controls (NC), assessed with Dunnett’s post hoc tests (α = 0.05); ns stands for not significant; *, *p* < 0.05; **, *p* < 0.01; ***, *p* < 0.001; ****, *p* < 0.0001. (**D**) Representative images of inoculated fruits at the end of the assayed period.

To explore the efficacy of the *E. amylovora* phage cocktails against *E. amylovora* strains from countries other than Spain, their performance against the French *E. amylovora* reference strain CFBP 1430 was also tested (Figure 5). The assays in a liquid medium showed that all the phage mixes were able to limit *E. amylovora* growth to OD_600nm_ values under those of the untreated controls (*p* < 0.0001) (Figure 5A). Among the assayed phage cocktails, mixes 2–4 kept the *E. amylovora* populations at levels close to the initial ones throughout the assay. However, as discussed above, mix 1 delayed the emergence of a subpopulation of phage-resistant host cells for about 7 h, which grew to OD_600nm_ values which were half of those reached by the controls towards the end of the experiment. These observations do not necessarily guarantee poor outcomes regarding the phage mix’s biocontrol activity.

The development of phage-resistance in other bacterial pathogens has been shown to trigger physiological changes in the resistant bacteria with trade-offs in terms of virulence, growth rates, and other fitness-related traits [79,80]. The delayed occurrence of phage-resistant cells and lower pathogen cell numbers in the presence of the phages might also influence the host cell’s overall ability to thrive in the plant tissues and cause infections. Linked to this, despite the results observed in vitro (Figure 5A), the assay with detached fruits revealed that phage mix 1 was as effective or even more effective in controlling the disease than the other three mixes, reaching control efficacy values of 66.7% (*p* < 0.0001) (Figure 5A) and reducing symptom severity to around 13.9% compared to the 100% severity developed by the pathogen inoculated alone (*p* < 0.0001) when applied preventively (Figure 5C,D).

The other *E. amylovora* phage mixes also affected the onset of symptoms, symptom development, and disease severity when assayed in the detached fruits. Out of the three remaining phage mixes (mixes 2–4), only mix 2 achieved a significant disease control efficacy of around 33% when applied preventively (*p* = 0.0132) (Figure 5B). 

None of the mixes provided effective control when co-inoculated with the pathogen. After being applied preventively, mixes 2–4 reduced symptom severity to 30.3–52.8% (*p* ≤ 0.0009). However, unlike mix 1, these mixes also achieved a significant reduction in symptom severity when co-inoculated with the pathogen, reaching percentages of 58.3–69.4% compared to the 100% observed in the controls (*p* ≤ 0.0428) (Figure 5C,D).

The assays with immature fruits performed in this work revealed that one single preventive application of some of the Mediterranean *E. amylovora* phages or their combinations not only delayed the onset of fire blight symptoms but also reduced the disease severity, suggesting their biocontrol potential in Spain and other countries. Given that, so far, there are no registered phage cocktails available in EU countries for plant pathogenic bacteria, including *E. amylovora* [80], this study is the first to design European phage cocktails against the fire blight pathogen. 

In the USA, there is a commercial phage-based product registered for the control of fire blight, called AgriPhage-Fire blight [25,80,81], which must be constantly improved due to the evolutionary nature of phages and their high specificity. Svircev’s group also developed, in Canada, a method of fire blight control based on the combined use of an antagonist of *E. amylovora*, the bacterium *P. agglomerans*, as a carrier of phages of this pathogen able to infect and lyse both bacterial species [23]. This method has required a recent optimization of the formulation and spray-drying protocol of *P. agglomerans* to improve the feasibility of industrial-scale production [82]. Recently, an evaluation of the efficacy of North American *E. amylovora* phages and the commercial AgriPhage product for fire blight management in four locations in the USA has shown that disease control ranged from 0.0 to 82.7%, obtaining the highest efficacy with the streptomycin control treatment [21]. This antibiotic is the most effective in reducing populations of the fire blight pathogen [83], but the emergence of resistant strains challenges its continued use [12,84]. Further, antibiotics are banned in EU countries [10] to reduce bacterial resistance development and antibiotic residues in food and the environment. Cupric compounds show relative efficacy in preventing fire blight infections. Still, their continuous application can cause fruit russeting and the selection of copper-resistant strains of *E. amylovora* and induce entry into the viable but non-culturable state (VBNC) of this pathogen [85]. The use of copper compounds is being increasingly restricted in the EU [10] due to their phytotoxicity and accumulation in the environment. Thus, bacteriophages can be an alternative and/or constitute additional biotools for integrated fire blight management programs. 

It is also worth mentioning that, although some phages or commercial phage products against the fire blight pathogen have demonstrated efficacy against some strains, their efficacy seems to be highly dependent on environmental conditions. Moreover, although *E. amylovora* is a highly homogeneous species, a great variation in the efficacy of different phages has been described depending on the strain of the pathogen tested [24,66,67,84]. Therefore, it is important to continue searching for new bacteriophages that can control fire blight outbreaks caused by different *E. amylovora* strains in a range of environmental settings. Further progress in optimizing their use by designing formulations and application methods that increase the survival of phages and/or their carrier bacteria in open environments is still needed [21,82]. 

### 3.5. Mediterranean E. amylovora Phages Are Myoviruses

**Figure 6:**
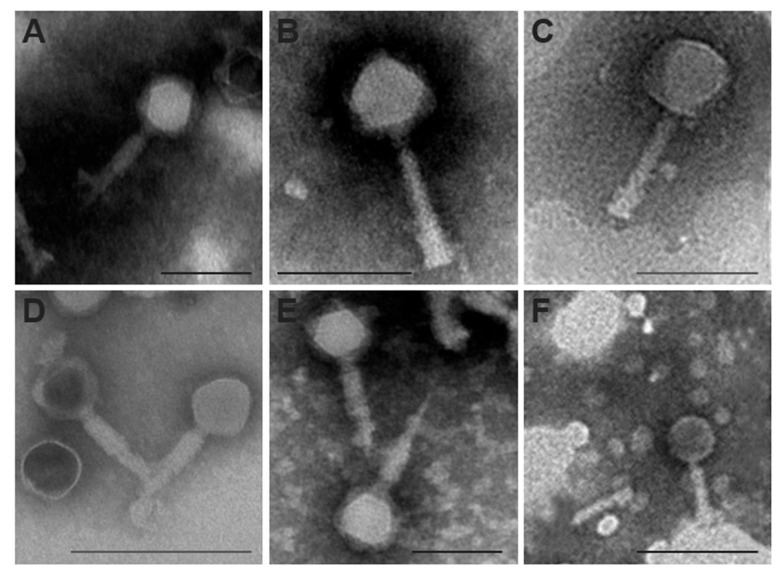
Transmission electron micrographs displaying the morphology of negatively stained Mediterranean *Erwinia amylovora* bacteriophages. (**A**) Phage ϕ6 magnified ×33,000; (**B**) phage ϕ21 magnified ×50,000; (**C**) phage ϕ24 magnified ×45,000; (**D**) phage ϕ25 magnified ×33,000; (**E**) phage ϕ27 magnified ×33,000; and (**F**) phage ϕ28 magnified ×22,000. Scale bars represent 100 nm in (**A**–**C**,**E**) and 200 nm in (**D**,**F**).

The six bacteriophages observed by transmission electron microscopy showed similar morphologies, with icosahedral heads between 60 and 78 nm in diameter, with a collar and long contractile tails between 93 and 102 nm (Figure 6). Phage ϕ6 (Figure 6A) has a capsid of 61.11 ± 5.06 nm and a tail of 93.02 ± 3.31 nm. The capsid of phage ϕ21 (Figure 6B) measures 59.82 ± 3.98 nm and its tail 94.56 ± 7.45 nm. Phage ϕ24 (Figure 6C) presents a capsid of 69.28 ± 5.32 nm and a tail of 94.22 ± 4.53 nm in length. Phage ϕ25 (Figure 6D) has a capsid of 63.85 ± 5.00 nm and a tail of 94.23 ± 9.23 nm. 

Phage ϕ27 (Figure 6E) displays a capsid of 63.46 ± 4.62 nm and a tail of 101.92 ± 4.62 nm. Finally, phage ϕ28 (Figure 6F) is the largest, with a capsid of 78.63 ± 7.41 nm in diameter and tails of 102.77 ± 5.4 nm in length. 

Following the classification model by Tolstoy et al. [86], distinctive features of myovirus (former *Myoviridae* family) include contractile, more or less rigid, and long (80–455 nm) tails separated from the head by a collar. According to the measurements and micrographs of the bacteriophages in our study, visualized with a transmission electron microscope (Figure 6), the six Mediterranean *E. amylovora* phages could be morphologically identified as myoviruses, from the present class of *Caudoviricetes,* in accordance with a recent taxonomy update of the ICTV bacterial viruses subcommittee [87].

### 3.6. Mediterranean E. amylovora Bacteriophages Are Members of the Genus Kolesnikvirus

The sequencing of the genomes from phages ϕ6, ϕ21, ϕ24, ϕ25, ϕ27, and ϕ28 (hereinafter referred to as vEam_PM_6, vEam_PM_21, vEam_S_24, vEam_W_25, vEam_PM_27, and vEam_W_28, respectively, where PM stands for plant material, S stands for soil, and W stands for water), all of them isolated in the Mediterranean settings, resulted in the assembly of six genomes ranging in size from 84,686 to 84,690 nt, with a consistent GC content of 43.41% in all cases. The annotation of the ORFs in these six genomes revealed a consistent coding of 117 in all cases. Additionally, analyses searching for other conserved domains of interest indicated that they do not encode any new endolysins, holins, or depolymerases, based on the data available in December 2023. PhageAI classified the six Mediterranean *E. amylovora* bacteriophages as virulent or lytic phages with a predicted value of 100%.

The BLASTn analysis performed with the six new *E. amylovora* phage genomes revealed a striking similarity of 99.8% between the six phages selected for this study and the bacteriophage species *Kolesnikvirus* M–M7 (subfamily *Ounavirinae*). 

**Figure 7:**
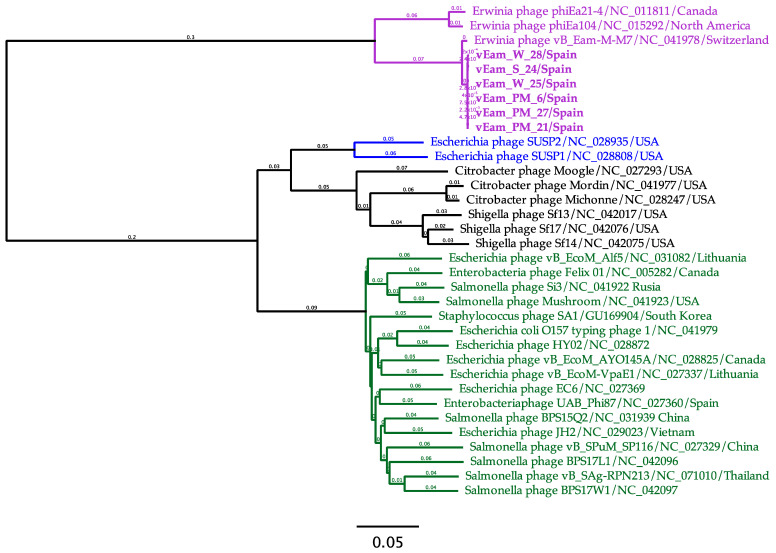
Phylogenetic proteomic tree of Mediterranean *Erwinia amylovora* bacteriophages (vEam_PM_6, vEam_PM_21, vEam_S_24, vEam_W_25, vEam_PM_27, and vEam_W_28) with a selection of closely related phages from other sources and continents, using the ViPTree server with a clustering distance selection of ≥0.05. The phage genus is indicated using the following colors: *Koleniskvirus* (purple), *Suspvirus* (blue), *Mooglevirus* (black), and *Felixounavirus* (green).

The similarity of these selected phages to other 28 RefSeq genomes within their respective genera was further evaluated through a phylogenetic analysis. The outcomes consistently demonstrated that the newly isolated phages clustered effectively within the *Kolesnikvirus* genus, specifically within the *Kolesnikvirus* M7 species cluster. Furthermore, the analysis successfully highlighted the appropriate clustering of species within the *Moglevirus*, *Suspvirus*, and *Felixounavirus* genera (Figure 7).

**Figure 8:**
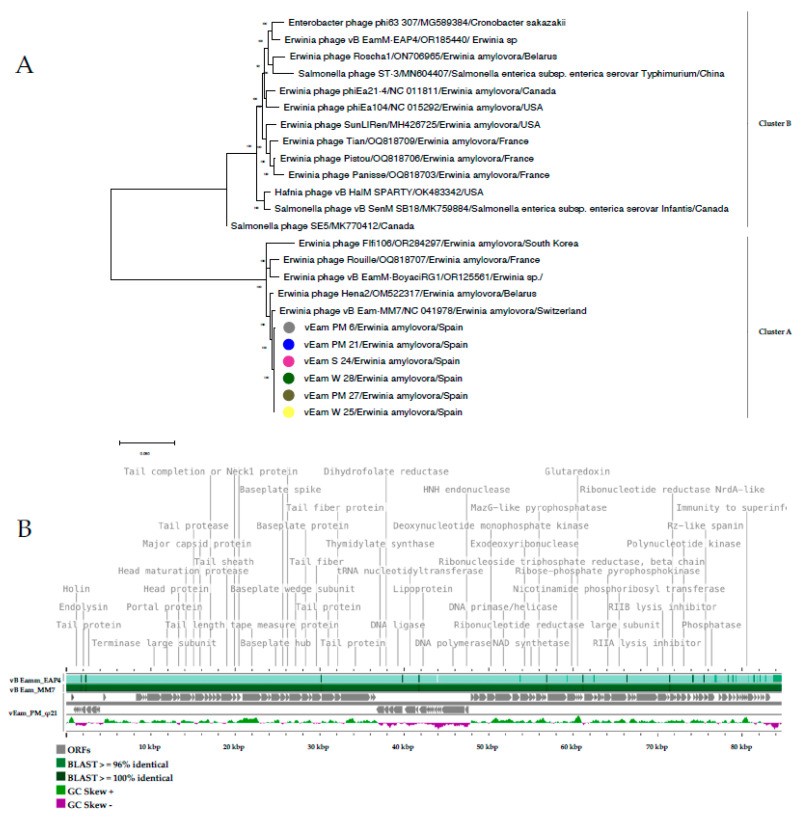
Comparative analysis carried out with the complete genomes of the six Mediterranean *Erwinia amylovora* phages and the two species within the genus *Kolesnikvirus*. (**A**) Maximum likelihood phylogenetic tree, including Mediterranean *E. amylovora* phages, constructed by MEGA X using the best substitution model, general time reversible with a G (0.20) + I (43.8% sites) parameter, with 32 complete phage genomes from the *Kolenskivirus* species: *Kolesnikvirus* M7 (Clade A) and *Kolesnikvirus* Ea214 (Clade B). Phage isolate name, accession numbers, host, and origin are indicated. The scale bar shows the number of substitutions per site. The bootstrap percentages (500 replicates) are indicated on the branches. (**B**) Genome map of the Mediterranean *E. amylovora* phage vEam_PM_21 compared by BLASTn (features by percent identity are indicated) with genome species *Kolesnikvirus* Ea214 (vB EamM EAP4 (OR185440)) and *Kolesnikvirus* M7 (vB Eam_MM7 (NC041978)). The Open Reading Frames (ORFs) are indicated in a grey color and the GC Skew score in green and purple colors.

Given that the results observed in the analysis of the proteomic tree revealed that the new phages described in this study belong to the *Kolesnikvirus* genus, a comparative analysis was conducted with all the complete genomes of the two species described in this genus (*Kolesnikvirus* Ea214 (KEa214) and *Kolesnikvirus* M7 (KM7)) (Figure 8A).

The results confirmed that the new Mediterranean phages of *E. amylovora* described in this study cluster with isolates from the KM7 species. Interestingly, in this analysis, it can be observed that phages with *E. amylovora* as a specific host only belong to the clade of the KM7 species, while the clade of the KEa214 species groups isolates which are capable of infecting both *E. amylovora* and other bacteria such as *Salmonella enterica* and *Hafnia alvei*. These results reinforce those obtained in the host range assays (Table 3) and suggest that isolates from the KM7 species may be more specific to *E. amylovora* than those of the KEa214 species. Therefore, these isolates could potentially be a suitable and safe tool to control *E. amylovora* without altering the natural microbiome of the host plant and the surrounding environment.

The comparative analyses of the complete genomes revealed that the six Mediterranean phages encode up to 117 ORFs, of which 47 have a known function, consistent with the vB Eam_MM7 isolate (NC041978). The number of polymorphisms between the Mediterranean phages and the reference genome NC041978 ranged from 398 to 418 nucleotides. These polymorphisms were predominantly located in intergenic regions and in some hypothetical proteins (Figure 8B), where mutations did not involve changes in the polarity and/or hydrophobicity of amino acids which could make changes in the protein structure encode.

Finally, in this analysis, based on complete genomes, another noteworthy result was observed: the new *E. amylovora* phage isolates from Mediterranean settings show a significant similarity to other *E. amylovora* phages isolated in Belarus (OM522317), France (OQ818707), Switzerland [35], and South Korea [88]. This finding suggests that phage species within this clade, in addition to potentially being specific to *E. amylovora*, exhibit a low degree of genetic divergence, similar to what is observed in the pangenome of *E. amylovora*. This genetic similarity suggests that the new Mediterranean *E. amylovora* phages could be of interest in the application of phage therapy for preventive control of *E. amylovora* in Spain and other countries.

## 4. Conclusions

In the present study, bacteriophages isolated in Mediterranean environments and characterized *in vitro* and *ex vivo* were able to efficiently and specifically infect and lyse *E. amylovora* strains from Spain and other countries. The phages are myoviruses belonging to the *Caudoviricetes* class and *Kolesnikvirus* genus. The biocontrol evaluation of selected phages and/or their combinations in *E. amylovora*-inoculated immature fruits revealed the potential of their preventive application in delaying the appearance of fire blight symptoms and reduce the severity of the disease in the host. Therefore, this study sets up preliminary steps towards designing bacteriophage cocktails effective against fire blight disease in the field, with these being the first cocktails of European phages of *E. amylovora* with successful biocontrol activity in plant material. Further work is still required to assess their biocontrol capacity under greenhouse and field conditions.

## Figures and Tables

**Table 1:** *Erwinia amylovora* strains used.

*E. amylovora* Strain Code	Host	Geographical Origin	Year
Spanish			
UPN ^1^ 527	*Malus x domestica*	Navarra	1997
IVIA ^2^ 1526.6	*Cotoneaster* sp.	Guipúzcoa	1996
IVIA 1554	*Crataegus* sp.	Segovia	1996
IVIA 1596	*Pyrus* sp.	Spain	1996
IVIA 1614.1	*Pyracantha* sp.	Spain	1996
IVIA 1614.2	*Cotoneaster* sp.	Segovia	1996
IVIA 1626.6	*M. x domestica*	Navarra	1996
IVIA 1892.1	*Pyrus* sp.	Guadalajara	1998
UV ^3^ P3P2AA1	*Pyrus communis*	Valencia *	2018
UV P4P2AA1	*P. communis*	Valencia	2018
UV P2P4TA1	*P. communis*	Valencia	2018
UV P2P4TA2.1	*P. communis*	Valencia	2018
UV P2EP4Tex	*P. communis*	Valencia	2018
UV P3P4TA1	*P. communis*	Valencia	2018
UV P3P4TA2	*P. communis*	Valencia	2018
**International**			
CFBP ^4^ 1430	*Crataegus oxyacantha*	France	1972
NCPPB ^5^ 311	*P. communis*	Canada	1952
Ea273	*Malus* sp.	USA	1971
CGJ2	*Malus* sp.	Serbia	2003

^1^ UPN: Universidad Pública de Navarra; ^2^ IVIA: Instituto Valenciano de Investigaciones Agrarias; ^3^ UV: Universitat de València; ^4^ CFBP: Collection Française de Bactèries Phytopathogènes; ^5^ NCPPB: National Collection of Plant Pathogenic Bacteria; and * former fire blight-affected zones in the province of Valencia.

**Table 2:** Bacterial species, including plant pathogens, used in the phage specificity assays.

Bacterial Species	Strain Code	Host	Geographical Origin
*Clavibacter michiganensis*	IVIA ^1^ 873	*Solanum lycopersicum*	Spain
*Dickeya* sp.	IVIA 4830	*S. lycopersicum*	Spain
*Pectobacterium atrosepticum*	IVIA 3447	*S. tuberosum*	Spain
*P. carotovorum*	IVIA 3902	*S. lycopersicum*	Spain
*Pseudomonas savastanoi* pv. *savastanoi*	IVIA 1628.3	*Olea europaea*	Spain
*Rhizobium radiobacter*	C58	*Prunus avium*	USA
*R. rhizogenes*	K84	Non-pathogenic	Australia
*Ralstonia solanacearum*	IVIA 1670	*S. tuberosum*	Spain
*Xanthomonas arboricola* pv. *pruni*	CITA ^2^ 33	*Prunus amygdalus*	Spain
*X. vesicatoria*	CECT ^3^ 792	Unknown	Israel
*Aeromonas hydrophila*	CECT 5173	Freshwater	France
*Alcaligenes faecalis*	CECT 928	Unknown	Unknown
*Bacillus cereus*	CECT 495	Chicken and turkey manure	Unknown
*Enterococcus faecalis*	CECT 481	Unknown	Unknown
*Escherichia coli*	CECT 101	Unknown	United Kingdom
*Klebsiella pneumonia*	CECT 143	Unknown	United States
*Kocuria rhizophila*	CECT 241	Soil	Unknown
*Pseudomonas fluorescens*	CECT 378	Pre-filter tanks, town water works	United Kingdom
*Proteus hauseri*	CECT 484	Unknown	Unknown
*Salmonella enterica* subsp. *enterica*	CECT 443	Human food poisoning	United Kingdom
*Serratia marcescens*	CECT 159	Unknown	Unknown
*Staphylococcus aureus*	CECT 4013	Bovine mammary gland	Unknown

^1^ IVIA: Instituto Valenciano de Investigaciones Agrarias; ^2^ CITA: Centro de Investigación y Tecnología Agroalimentaria de Aragón; and ^3^ CECT: Colección Española de Cultivos Tipo (Spanish Type Culture Collection).

**Table 3:** Host range of a selection of 28 Mediterranean bacteriophages against a collection of 19 *Erwinia amylovora* strains and 22 strains of other bacterial species, assessed by the spot test.

Host Strain Code	UV *E. amylovora* Bacteriophages																											
	ϕ1	ϕ2	ϕ3	ϕ4	ϕ5	ϕ6	ϕ7	ϕ8	ϕ9	ϕ10	ϕ11	ϕ12	ϕ13	ϕ14	ϕ15	ϕ16	ϕ17	ϕ18	ϕ19	ϕ20	ϕ21	ϕ22	ϕ23	ϕ24	ϕ25	ϕ26	ϕ27	ϕ28
*E. amylovora* UPN 527	+	+	+	+	+	+	+	+	+	+	+	+	+	+	+	+	+	+	+	+	+	+	+	+	+	+	+	+
*E. amylovora* IVIA 1526.6	+	+	+	+	+	+	+	+	+	+	+	+	+	+	+	+	+	+	+	+	+	+	+	+	+	+	+	+
*E. amylovora* IVIA 1554	+	+	+	+	+	+	+	+	+	+	+	+	+	+	+	+	+	+	+	+	+	+	+	+	+	+	+	+
*E. amylovora* IVIA 1596	-	-	+	-	-	+	+	+	W	-	-	W	W	-	-	-	W	W	-	W	+	W	-	-/+	W	+	-/+	-/+
*E. amylovora* IVIA 1614.1	+	+	W	+	+	+	+	+	+	-	+	+	+	+	+	-	W	+	+	+	+	+	+	+	+	W	+	+
*E. amylovora* IVIA 1614.2	+	+	+	-	+	+	+	+	+	+	+	+	W	+	+	+	+	+	+	+	+	+	+	+	+	W	-/+	+
*E. amylovora* IVIA 1626.6	W	W	-	W	W	W	-	-	-	W	-	-	W	-	-	W	W	-	-	-	-/+	-	-	-/+	-/+	W	W	-/+
*E. amylovora* IVIA 1892.1	+	+	+	+	+	+	+	+	+	+	+	+	+	+	-	+	+	+	-	-	+	-	-	-/+	+	+	-/+	-/+
*E. amylovora* UV P3P2AA1	W	-	W	W	-	+	+	+	+	-	-	-	-	W	-	W	W	W	W	W	+	-	-	W	+	+	W	W
*E. amylovora* UV P4P2AA1	+	-	+	+	+	+	+	+	+	+	-	-	+	+	-	+	+	-	+	+	+	-	+	+	+	+	+	+
*E. amylovora* UV P2P4TA1	+	-	+	-	+	+	+	+	+	+	-	-	+	+	-	+	+	-	+	-	+	+	+	-/+	+	+	-/+	-/+
*E. amylovora* UV P2P4TA2.1	+	+	W	+	+	W	W	W	W	W	-	-	+	+	W	-	-	-	-	-	W	W	-	-/+	+	W	+	-/+
*E. amylovora* UV P2exP4T	+	+	+	+	+	+	+	+	+	+	-	-	+	+	-	+	+	-	W	W	+	-	W	W	+	+	+	W
*E. amylovora* UV P3P4TA1	+	+	-	+	+	W	W	W	-	+	+	+	+	+	+	+	+	+	+	+	+	+	+	+	+	W	+	+
*E. amylovora* UV P3P4TA2	+	+	W	+	+	W	W	W	W	+	+	+	+	W	+	+	+	+	+	+	W	+	+	+	W	+	+	+
*E. amylovora* CFBP 1430	-	-	-	W	-	W	-	W	-	W	+	+	-	+	+	W	W	+	+	+	-/+	+	+	+	-/+	-	-/+	W
*E. amylovora* NCPPB 311	W	W	+	W	W	+	+	+	+	+	+	+	+	+	+	+	W	+	W	W	+	W	W	W	+	+	W	W
*E. amylovora* GJ-2	+	+	+	+	+	+	+	+	W	W	W	W	+	+	W	W	W	W	W	W	+	W	W	W	+	W	+	W
*E. amylovora* 273	-	-	-	-	-	W	-	-	-	W	-	W	-	-	W	W	-	W	W	W	W	W	-	W	-	W	-	-
*C. michiganensis* IVIA 873	-	-	-	-	-	-	-	-	-	-	-	-	-	-	-	-	-	-	-	-	-	-	-	-	-	-	-	-
*Dickeya* sp. IVIA 4830	-	-	-	-	-	-	-	-	-	-	-	-	-	-	-	-	-	-	-	-	-	-	-	-	-	-	-	-
*P. atrosepticum* IVIA 3447	-	-	-	-	-	-	-	-	-	-	-	-	-	-	-	-	-	-	-	-	-	-	-	-	-	-	-	-
*P. carotovorum* IVIA 3902	-	-	-	-	-	-	-	-	-	-	-	-	-	-	-	-	-	-	-	-	-	-	-	-	-	-	-	-
*P. savastanoi* IVIA 1628.3	-	-	-	-	-	-	-	-	-	-	-	-	-	-	-	-	-	-	-	-	-	-	-	-	-	-	-	-
*R. radiobacter* C58	-	-	-	-	-	-	-	-	-	-	-	-	-	-	-	-	-	-	-	-	-	-	-	-	-	-	-	-
*R. rhizogenes* K84	-	-	-	-	-	-	-	-	-	-	-	-	-	-	-	-	-	-	-	-	-	-	-	-	-	-	-	-
*R. solanacearum* IVIA 1670	-	-	-	-	-	-	-	-	-	-	-	-	-	-	-	-	-	-	-	-	-	-	-	-	-	-	-	-
*X. arboricola* pv. *pruni* CITA 33	-	-	-	-	-	-	-	-	-	-	-	-	-	-	-	-	-	-	-	-	-	-	-	-	-	-	-	-
*X. vesicatoria* CECT 792	-	-	-	-	-	-	-	-	-	-	-	-	-	-	-	-	-	-	-	-	-	-	-	-	-	-	-	-
*A. hydrophila* CECT 5173	-	-	-	-	-	-	-	-	-	-	-	-	-	-	-	-	-	-	-	-	-	-	-	-	-	-	-	-
*A. faecalis* CECT 928	-	-	-	-	-	-	-	-	-	-	-	-	-	-	-	-	-	-	-	-	-	-	-	-	-	-	-	-
*B. cereus* CECT 495	-	-	-	-	-	-	-	-	-	-	-	-	-	-	-	-	-	-	-	-	-	-	-	-	-	-	-	-
*E. faecalis* CECT 481	-	-	-	-	-	-	-	-	-	-	-	-	-	-	-	-	-	-	-	-	-	-	-	-	-	-	-	-
*E. coli* CECT 101	-	-	-	-	-	-	-	-	-	-	-	-	-	-	-	-	-	-	-	-	-	-	-	-	-	-	-	-
*K. pneumoniae* CECT 143	-	-	-	-	-	-	-	-	-	-	-	-	-	-	-	-	-	-	-	-	-	-	-	-	-	-	-	-
*K. rhizophila* CECT 241	-	-	-	-	-	-	-	-	-	-	-	-	-	-	-	-	-	-	-	-	-	-	-	-	-	-	-	-
*P. fluorescens* CECT 378	-	-	-	-	-	-	-	-	-	-	-	-	-	-	-	-	-	-	-	-	-	-	-	-	-	-	-	-
*P. hauseri* CECT 484	-	-	-	-	-	-	-	-	-	-	-	-	-	-	-	-	-	-	-	-	-	-	-	-	-	-	-	-
*S. enterica* CECT 443	-	-	-	-	-	-	-	-	-	-	-	-	-	-	-	-	-	-	-	-	-	-	-	-	-	-	-	-
*S. marcescens* CECT 159	-	-	-	-	-	-	-	-	-	-	-	-	-	-	-	-	-	-	-	-	-	-	-	-	-	-	-	-
*S. aureus* CECT 4013	-	-	-	-	-	-	-	-	-	-	-	-	-	-	-	-	-	-	-	-	-	-	-	-	-	-	-	-
*E. amylovora* strains lysed (%)	84	68	79	79	79	100	84	90	79	74	58	68	84	84	63	84	90	74	80	79	90	74	68	74	84	95	68	68
Non-*E. amylovora* species lysed (%)	0	0	0	0	0	0	0	0	0	0	0	0	0	0	0	0	0	0	0	0	0	0	0	0	0	0	0	0

+, lysis by spot test on solid medium; -, no lysis; W, weak lysis; and -/+, no lysis by spot test on solid medium/lysis in liquid medium with selected phages.

## Data Availability

The genome sequences of the six Mediterranean *Erwinia amylovora* bacteriophages (vEam_PM_6, vEam_PM_21, vEam_S_24, vEam_W_25, vEam_PM_27, and vEam_W_28) were deposited at GenBank (PP079182, PP079183, PP079184, PP079185, PP079186, and PP079187).

## Supplementary Materials

The following supporting information can be downloaded at https://www.mdpi.com/article/10.3390/biology13030176/s1, Table S1: Origin of a selection of 28 Mediterranean bacteriophages with lytic activity against *Erwinia amylovora* (Ea).
