# Supplementary material for: First European Erwinia amylovora Lytic Bacteriophage Cocktails Effective in the Host: Characterization and Prospects for Fire Blight Biocontrol
Source: Biology (Basel). 2024 Mar 8;13(3):176. doi: 10.3390/biology13030176 (PMC10967764; doi:10.3390/biology13030176)
Supplement: Supplementary file 1 [file biology-13-00176-s001.zip › Supplementary_info Table S1.pdf]

Supplementary Table S1. Origin of a selection of 28 Mediterranean bacteriophages with lytic activity against *Erwinia amylovora* (Ea).

Phage ID[a]	Sample material	Location and Plot[b]	Spanish region	Year	Ea strain lysed in the first phage isolation
UV_Eaϕ1	Plant material washings (pear trees)	Location 1, Plot 2	Comunidad Valenciana	2018	UPN 527
UV_Eaϕ2	Plant material processed by crushing (pear trees)	Location 1, Plot 2	Comunidad Valenciana	2018	UPN 527
UV_Eaϕ3	Plant material washings (pear trees)	Location 1, Plot 2	Comunidad Valenciana	2018	UPN 527
UV_Eaϕ4	Plant material washings (pear trees)	Location 1, Plot 3	Comunidad Valenciana	2018	UPN 527
UV_Eaϕ5	Plant material washings (pear trees)	Location 1, Plot 2	Comunidad Valenciana	2018	UPN 527
**UV_Eaϕ6**	Plant material processed by crushing (pear trees)	Location 1, Plot 3	Comunidad Valenciana	2018	IVIA 1526.6
UV_Eaϕ7	Plant material processed by crushing (pear trees)	Location 1, Plot 3	Comunidad Valenciana	2018	IVIA 1526.6
UV_Eaϕ8	Plant material processed by crushing (pear trees)	Location 1, Plot 2	Comunidad Valenciana	2018	IVIA 1526.6
UV_Eaϕ9	Plant material processed by crushing (pear trees)	Location 1, Plot 4	Comunidad Valenciana	2018	IVIA 1554
UV_Eaϕ10	Soil under symptomatic pear trees	Location 1, Plot 2	Comunidad Valenciana	2018	IVIA 1554
UV_Eaϕ11	Plant material washings (pear trees)	Location 1, Plot 2	Comunidad Valenciana	2018	IVIA 1554
UV_Eaϕ12	Plant material processed by crushing (pear trees)	Location 1, Plot 2	Comunidad Valenciana	2018	IVIA 1554
UV_Eaϕ13	Plant material washings (pear trees)	Location 1, Plot 2	Comunidad Valenciana	2018	IVIA 1614.1
UV_Eaϕ14	Plant material processed by crushing (pear trees)	Location 1, Plot 4	Comunidad Valenciana	2018	IVIA 1614.2
UV_Eaϕ15	Plant material processed by crushing (pear trees)	Location 1, Plot 2	Comunidad Valenciana	2018	IVIA 1614.2
UV_Eaϕ16	Soil under symptomatic pear trees	Location 1, Plot 2	Comunidad Valenciana	2018	IVIA 1614.2
UV_Eaϕ17	Plant material washings (pear trees)	Location 1, Plot 4	Comunidad Valenciana	2018	IVIA 1614.2
UV_Eaϕ18	Plant material processed by crushing (pear trees)	Location 1, Plot 4	Comunidad Valenciana	2018	IVIA 1614.2
UV_Eaϕ19	Soil under symptomatic pear trees	Location 1, Plot 4	Comunidad Valenciana	2018	IVIA 1892.1
UV_Eaϕ20	Plant material processed by crushing (pear trees)	Location 1, Plot 3	Comunidad Valenciana	2018	IVIA 1892.1
**UV_Eaϕ21**	Plant material washings (pear trees)	Location 1, Plot 2	Comunidad Valenciana	2018	IVIA 1892.1
UV_Eaϕ22	Plant material processed by crushing (pear trees)	Location 1, Plot 2	Comunidad Valenciana	2018	IVIA 1892.1
UV_Eaϕ23	Plant material processed by crushing (pear trees)	Location 1, Plot 2	Comunidad Valenciana	2018	IVIA 1892.1
**UV_Eaϕ24**	Soil under symptomatic pear trees	Location 1, Plot 3	Comunidad Valenciana	2018	IVIA 1892.1
**UV_Eaϕ25**	Water source nearby a plot with symptomatic pear trees	Location 2, Plot 4	Comunidad Valenciana	2018	IVIA 1614.1
UV_Eaϕ26	Water source nearby a plot with symptomatic pear trees	Location 2, Plot 4	Comunidad Valenciana	2018	IVIA 1614.1
**UV_Eaϕ27**	Plant material processed by crushing (pear trees)	Location 2, Plot 4	Comunidad Valenciana	2018	IVIA 1892.1
**UV_Eaϕ28**	Water source nearby a plot with symptomatic pear trees	Location 2, Plot 4	Comunidad Valenciana	2018	IVIA 1892.1

[a] Bold letters, phages selected to be mixed in phage cocktails.

[b] Former fire-blight affected plots of pear trees in two municipalities in the Comunidad Valenciana, Spain, under official control.
